# A fatal complication after repair of post-infarction ventricular septal rupture: heparin-induced thrombocytopenia with thrombosis

**DOI:** 10.5830/CVJA-2015-001

**Published:** 2015

**Authors:** Yunus Nazli, Necmettin Colak, Mehmet Fatih Alpay, Omer Cakir, Bora Demircelik, Kerim Cagli

**Affiliations:** Department of Cardiovascular Surgery, Faculty of Medicine, University of Turgut Ozal, Ankara, Turkey; Department of Cardiovascular Surgery, Faculty of Medicine, University of Turgut Ozal, Ankara, Turkey; Department of Cardiovascular Surgery, Faculty of Medicine, University of Turgut Ozal, Ankara, Turkey; Department of Cardiovascular Surgery, Faculty of Medicine, University of Turgut Ozal, Ankara, Turkey; Department of Cardiology, Faculty of Medicine, University of Turgut Ozal, Ankara, Turkey; ---

**Keywords:** heparin, thrombocytopenia, thrombosis, post-infarction ventricular septal rupture

## Abstract

Heparin-induced thrombocytopenia (HIT) is a rare but potentially devastating and life-threatening complication from using heparin. HIT not only causes thrombocytopenia, but it also carries an increased risk for fatal thrombotic complications. In this report, we describe the case of a patient in whom fatal HIT developed after successful surgical repair of a posterior post-infarction ventricular septal rupture with cardiopulmonary bypass.

## Abstract

Heparin-induced thrombocytopenia (HIT) is a rare but potentially devastating and life-threatening complication of heparin therapy. HIT not only causes thrombocytopenia, but it also carries an increased risk for both arterial and venous thrombotic complications, despite the administration of heparin as an anticoagulating agent.^1^

HIT is associated with antibodies to a complex of heparin–platelet factor 4 (H-PF4). HIT-associated antibodies are generally detected after open-heart surgery.^2^,^3^ Post-infarction ventricular septal rupture (PI-VSR) following acute myocardial infarction has a high mortality rate and surgical repair also presents a high risk of mortality.

In this report, we describe the case of a patient in whom fatal HIT developed after successful surgical repair of a posterior PI-VSR on cardiopulmonary bypass (CPB). This is rare, and a limited number of cases have been reported following surgical repair of a PI-VSR.

## Case report

A 74-year-old man presented with chest pain to a local hospital, from where he was transferred to our institution, with a PI-VSR. He had been anticoagulated with unfractioned heparin (UFH) (1 000 IU/h daily) for three days since the myocardial infarction (MI) had occurred.

**Figure 1.**
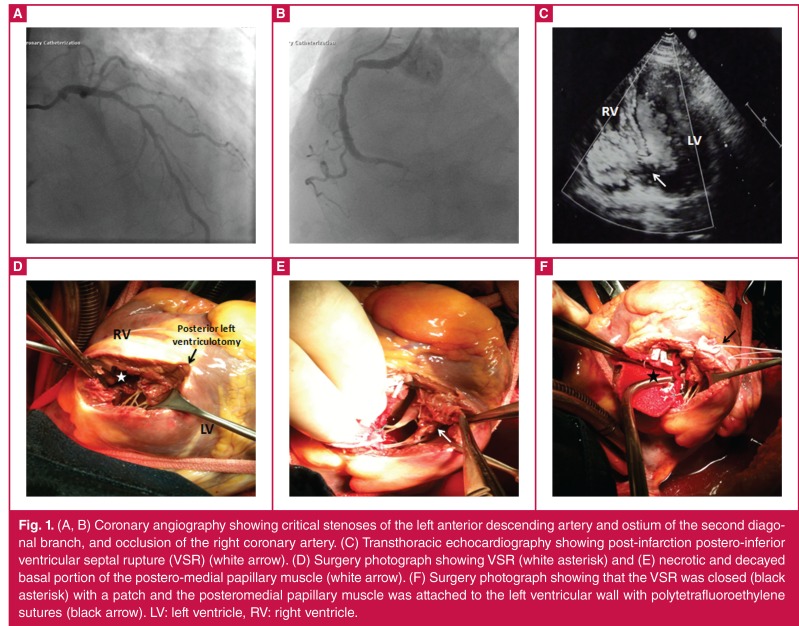
(A, B) Coronary angiography showing critical stenoses of the left anterior descending artery and ostium of the second diagonal branch, and occlusion of the right coronary artery. (C) Transthoracic echocardiography showing post-infarction postero-inferior ventricular septal rupture (VSR) (white arrow). (D) Surgery photograph showing VSR (white asterisk) and (E) necrotic and decayed basal portion of the postero-medial papillary muscle (white arrow). (F) Surgery photograph showing that the VSR was closed (black asterisk) with a patch and the posteromedial papillary muscle was attached to the left ventricular wall with polytetrafluoroethylene sutures (black arrow). LV: left ventricle, RV: right ventricle.

On admission, his heart rate was 92 beats/min, blood pressure was 90/50 mmHg and weight was 85 kg. Physical examination showed a systolic murmur at the left sternal border. Cardiac catheterisation was performed, during which a single intravenous dose of 2 500 units of heparin was administered. Coronary angiography showed critical stenoses in the mid segment of the left anterior descending artery and ostium of the second diagonal branch, and occlusion in the distal segment of the right coronary artery (Fig. 1A, B). The time between the onset of acute MI and surgery was three days.

Transthoracic echocardiography revealed poor left ventricular wall motion and a large postero-inferior ventricular septal rupture (Fig. 1C). To prevent cardiogenic shock, intra-aortic balloon pump assistance was initiated and anticoagulation was continued with a heparin infusion of 500 IU/h over three hours.

Thereafter, the patient was taken to the operating room for emergency surgery because of haemodynamic deterioration. After induction of general anaesthesia, transoesophageal echocardiography (TEE) was performed to evaluate the repair of the VSR. A median sternotomy was performed. Cardiopulmonary bypass was instituted with ascending aortic and bicaval venous cannulation. Heparin (300 U/kg) was given to obtain an activated clotting time of more than 400 s.

Firstly, a longitudinal transinfarction incision was made in the left ventricular myocardium parallel to and 1 cm away from the posterior descending artery. The post-infarction VSR was closed with a double velour fabric polyester patch (Bard® Debakey®, IMPRA, Inc) using a 3-0 polypropylene suture (Ethicon, Inc, Somerville, NJ) with a teflon pledget through the left ventricle (Fig. 1D).

The posteromedial papillary muscle was carefully inspected and the basal portion of the posteromedial papillary muscle appeared to be necrotic (Fig. 1E). The decayed base of the posteromedial papillary muscle was attached to the left ventricular wall using two interrupted mattress 4-0 polytetrafluoroethylene (goretex) sutures with a teflon pledget (Fig. 1F). The posterior left ventriculotomy was closed in two layers over two teflon felt strips using 2-0 polypropylene sutures (Ethicon, Inc, Somerville, NJ).

CABG was then performed with sequential grafting of the saphenous vein to the left anterior descending artery and second diagonal branch. Peri-operative transoesophageal echocardiography demonstrated well-preserved left ventricular wall contraction except for the infarcted area, with no evidence of residual leak.

Successful weaning from cardiopulmonary bypass was achieved with an intra-aortic balloon pump (IABP) and low-dose inotropic support. The patient tolerated the surgical procedure well, and his initial postoperative course was uneventful. He remained intubated for 13 hours in ICU with a 24- and 48-hour postoperative blood loss of 750 and 300 ml, respectively. For 48 hours during the immediate postoperative period, the patient was managed with intra-aortic balloon counter pulsation and small doses of inotropic drugs. He was weaned off IABP on the third postoperative day.

A total dose of 98 500 IU of heparin was given before (72 000 IU) and during (26 500 IU) cardiopulmonary bypass (CPB) at our hospital. Anticoagulation was reversed by protamine at the end of the operation. Following separation from bypass, the patient was given a total of six units of fresh frozen plasma and eight units of packed red cells.

**Figure 2.**
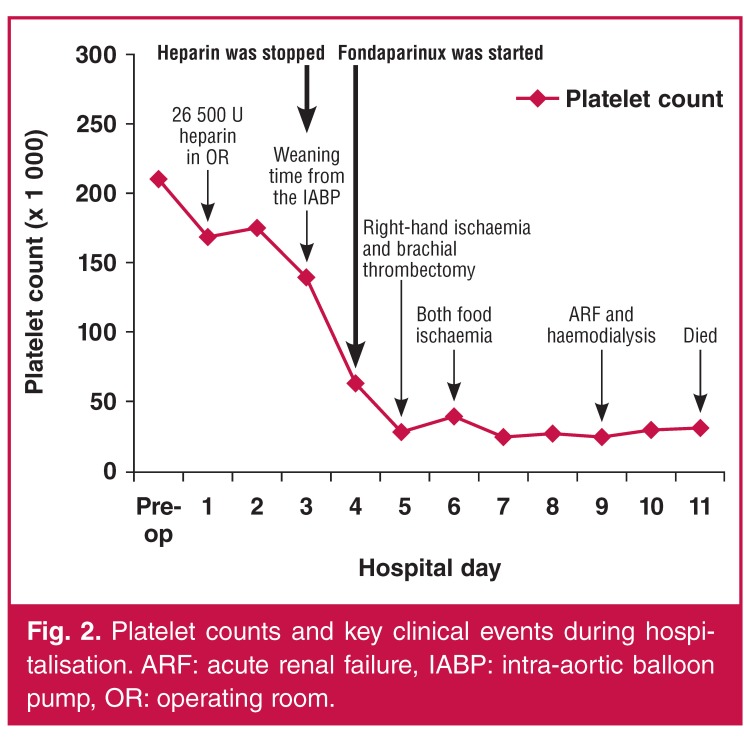
Platelet counts and key clinical events during hospitalisation. ARF: acute renal failure, IABP: intra-aortic balloon pump, OR: operating room.

Blood tests were done regularly and the platelet count was monitored daily. Laboratory findings on admission showed a normal platelet count (210 × 10^3^ cells/μl). Thrombocytopaenia developed postoperatively on day four with falls in platelet count of more than 50%, with a platelet level of 64 × 10^3^ cells/μl, from an initial admission count of 210 × 10^3^ cells/μl. The nadir platelet count was 25 × 10^3^ cells/μl on the seventh postoperative day (Fig. 2). Fig. 2 summarises the patient’s platelet counts and key clinical events during hospitalisation.

On postoperative day five, right-hand cyanosis was noted with absent radial pulses and was attributed to the presence of a right radial arterial catheter. The radial arterial catheter was removed the same day without improvement. Doppler ultrasound showed an occlusion of the radial artey and a patent ulnar artery. Also, a superficial venous thrombosis (cephalic and basilic vein) was detected in the right arm by Doppler ultrasound.

**Figure 3.**
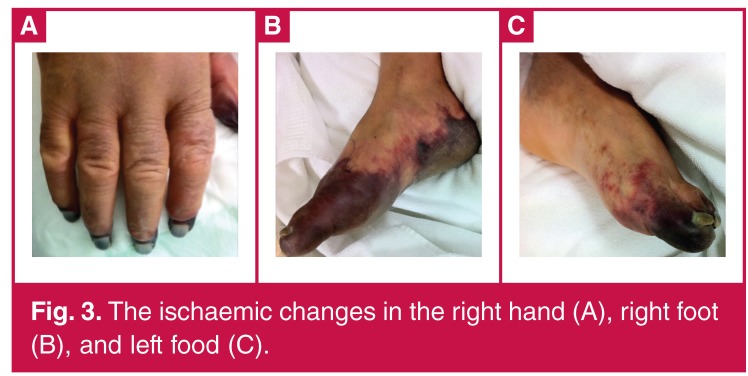
The ischaemic changes in the right hand (A), right foot (B), and left food (C).

On postoperative day six, ischaemic changes developed on the front of both feet (Fig. 3). The ischaemic changes in the right hand worsened from that of the previous day. Additionally, an occlusion of the right ulnar artery was detected by Doppler ultrasound. A brachial artery thrombectomy was performed three times on the same day because of increased ischaemic changes. The thrombectomy was not successful.

We suspected type 2 HIT. Heparin therapy was immediately discontinued on postoperative day three, including all intravenous fluids and lines. On the basis of the clinical symptoms, we used the ‘4 Ts’ clinical scoring system to test for the possibility of HIT, and found a high probability of heparin-induced thrombocytopenia.

A laboratory test was performed on postoperative day six, and a definitive diagnosis of HIT was made by serological test, confirming positive antibodies to the heparin–PF4 complexes with a slow turnaround time. Complete thrombophilic studies were unremarkable for other hypercoagulable conditions.

Anticoagulation was immediately started with fondaparinux, which is the only alternative anticoagulant agent in our country, at the recommended dose for these patients (7.5 mg/day, subcutaneous) on postoperative day four because of a fall in platelet count of more than 50%. The patient’s platelet count had not increased during therapy with fondaparinux after seven days.

The patient developed acute renal insufficiency requiring haemodialysis on postoperative day nine. Fondaparinux (2.5 mg) was also instilled directly into the dialysis circuit on dialysis days. On the 11th day postoperatively, the patient died of multiple organ failure despite intensive care.

## Discussion

Unfractionated heparin (UFH) is routinely used worldwide during CPB procedures and other various conditions for systemic anticoagulation.^4^ However, a small percentage of patients treated with UFH or low-molecular weight heparin (LMWH) suffer complications caused by side effects of the drug, the most serious of which is HIT.^1^ HIT is an adverse effect of the drug causing potentially fatal thrombotic or thromboembolic complications. HIT is a clinicopathological condition initiated with heparin exposure and characterised by a fall in the platelet count and paradoxical thrombophilia.^5^,^6^

HIT syndrome may be classified into two distinct subtypes based on differences in the pathophysiology and clinical features: type 1 and type 2. Type 1 HIT is non-immune and typically occurs as a fall in platelet count within the first two days after starting heparin. Platelet levels generally decrease by 10–20%.^7^ This condition usually resolves spontaneously without treatment or complications within days, even with continued heparin use. It is a non-immune-mediated disorder and appears to be due to a direct activation of the platelets by heparin, leading to platelet aggregation and, as a result, thrombocytopenia.

By contrast, the less common and more severe form, type 2 HIT is an immune-mediated disorder caused by antibody formation against the circulating H-PF4 complexes. This type can be associated with thrombotic or thromboembolic complications. It is also known as heparin-induced thrombocytopenia and thrombosis (HITT) and white clot syndrome due to platelet-rich arterial thrombosis.^5^ In most cases, thrombocytopenia develops on approximately the fifth day of initiation of heparin.^8^

The incidence of type 2 HIT is significantly higher after exposure to UFH versus LMWH (2.6 vs 0.2%).^9^ Surgical patients (especially cardiac surgery) are also more likely to develop HIT than medical patients.^5^

The incidence of HITT in patients who have undergone cardiac surgery has been estimated at between 0.12 and 1.3%.^10^ Cardiac surgical patients are at a greater risk for postoperative HITT due to several factors. First, most of these patients have had previous exposure to heparin for diagnostic, prophylactic and therapeutic purposes. Second, they are exposed to high-dose intra-operative heparin during CPB, and platelet activation is associated with surgery and CPB. Third, this exposure is usually continued in the postoperative period (either prophylactically or for flushing the lines).^11^

The common clinical presentation of HIT involves thrombocytopenia and thrombosis. Thrombocytopenia is the primary manifestation of HIT, but the degree and onset of the fall in platelet count may be variable. Type 1 HIT is often characterised by a fall in platelet count within one and four days after heparin exposure, with a nadir level of 100 000 cells/μl, spontaneous normalisation despite continued heparin use, and no other clinical sequelae.

On the other hand, type 2 HIT occurs within five to 10 days after the administration of heparin. The platelet counts fall more significantly by ≥ 50% or ≥ 100 000 cells/μl, with a median nadir of ~ 60 000 cells/μl.^7^ However, even when platelet counts in type 2 HIT are typically < 20 000/μl, spontaneous bleeding is uncommon. Thrombosis is the main contributor to morbidity and mortality associated with type 2 HIT, and HIT is fatal in an estimated 5–10% of patients, typically due to thrombotic or thromboembolic events.

Thrombosis may accompany thrombocytopenia in 30–60% of patients. Although thrombosis may occur in any vascular bed, venous thrombosis is more common than arterial thrombosis and often presents as deep-vein thrombosis or pulmonary embolism. However, arterial thrombosis can be predominant in cardiac and vascular surgical patients.^5^ Major complications of arterial thrombosis are acute limb ischaemia, stroke and acute myocardial infarction. Arterial thrombi are uncommonly seen in the renal, mesenteric or spinal arteries.^4^

The diagnosis of HIT is essentially made on the basis of clinical grounds and a decrease in platelet count in a patient receiving heparin, for which there are no obvious causes. Laboratory assays (frequently with slow turnaround times) play a supportive role and involve functional and non-functional tests.12 Most laboratory tests are not readily available in the acute setting.8

HIT should be suspected in the setting of absolute thrombocytopenia (platelet count < 150 000 cells/μl) as well as relative thrombocytopenia (fall in platelet count of at least 50% from baseline value). However, this syndrome should also be considered in patients with the unexplained development of new or progressive thrombosis while receiving a heparin product.

To aid in the diagnosis of HIT, a specific scoring system for clinical diagnosis, called the 4 Ts score was developed and validated by Lo *et al.*^13^ A score is calculated based on the following four categories: degree of thrombocytopenia, timing of onset of thrombocytopenia, clinical sequelae such as the development of venous or arterial thrombosis, and presence of other aetiologies of thrombocytopenia.^5^

Functional and immunological tests for HIT involve the platelet aggregation test (PAT), serotonin release assay (SRA), heparin-induced platelet aggregation (HIPA) test, the anti-HPF4 complex antibody enzyme-linked immunosorbent assays (ELISA), and flow cytometry studies.^8^ The sensitive (> 90%) but less specific (~ 71%) H-PF4 ELISA test is often used as a screening test, and the SRA (sensitivity and specificity 100 and 97%, respectively) can be performed as a confirmatory test, but is not universally available and utilised.^12^

The HIT-associated mortality rate in cardiac surgery patients is 35–42%.^7^ About 20% of patients developing HIT syndrome may require limb amputation because of peripheral arterial thrombosis. This syndrome is associated with a myriad of complications, including multiple organ systems: mesenteric ischaemia, renal insufficiency and stroke.^8^

Delays in the availability of diagnostic assay results frequently necessitate initiation of treatment for HIT based on clinical evaluation alone. When HIT with or without thrombosis is suspected postoperatively, the first step in treatment is immediate discontinuation of all heparin exposure, including heparin flushes and LMWH.^12^

In addition to heparin discontinuation, patients with either HIT with thrombosis or isolated HIT (type 2 HIT without thrombosis) require further treatment with an alternative anticoagulant agent. Heparin discontinuation alone is insufficient, because patients (even type 2 HIT without thrombosis) remain in a prothrombotic state. In light of the sustained thrombus propagation that occurs with HIT, current treatment is focused on reduction of thrombin generation via direct thrombin inhibition (e.g. bivalirudin, lepirudin, argatroban) or indirect factor Xa inhibition (e.g. danaparoid, fondaparinux).^5^

Prophylactic and therapeutic fondaparinux failed to prevent the development of and to treat HIT in our case, but we used fondaparinux because it is the only alternative anticoagulant agent in our country. Although successful results have been reported in the treatment of HIT with the use of fondaparinux, it was recommended as grade 2C at the 9th ACCP Conference on Antithrombotic Therapy and Prevention of Thrombosis.^14^

There are often plausible alternative explanations in critical patients with thrombocytopenia. CPB and the use of the IABP are clearly associated with thrombocytopenia. However, in our patient, the decreased platelet count was not attributed to these devices and conditions because there was no drop (> 50%) in platelet count within the first three postoperative days. It is also essential to differentiate HIT from other conditions causing thrombocytopenia, such as haemodilution, disseminated intravascular coagulation and sepsis. In our patient, these conditions were excluded by several examinations and laboratory tests.

## Conclusion

HIT is a clinicopathological syndrome in which one or more clinical events occur, usually thrombocytopenia or thrombosis. Patients undergoing cardiac surgery can be at risk of HIT in the early postoperative period, therefore daily platelet counts should be performed during this period. If signs of HIT develop in a patient receiving heparin, it must be stopped immediately and alternative anticoagulant agents started. In our case, we did not have success with fondaparinux as the alternative anticoagulant. Despite discontinuation of heparin and initiation of alternative anticoagulant agents, high morbidity and mortality rates are associated with HIT.
